# Neurobehavioral and Dopaminergic Dysfunctions Induced by Mixed Metal Exposure in C57BL/6 Mice

**DOI:** 10.5812/ijpr-158559

**Published:** 2025-03-02

**Authors:** Daeun Lee, Haesoo Kim, Sarita Pyatha, Kisok Kim

**Affiliations:** 1 College of Pharmacy, Keimyung University, Daegu, South Korea; 2 BK21 Four Center for Forensic Pharmaceutical Sciences, Keimyung University, Daegu, South Korea

**Keywords:** Aluminum, Lead, Mercury, Motor Activity, Locomotion, Dopamine, Corpus Striatum

## Abstract

**Background:**

Aluminum (Al), lead (Pb), and mercury (Hg) are major environmental pollutants, and a large population may be simultaneously exposed to these metals. However, studies on the potential neurobehavioral effects of mixed exposure to Al, Pb, and Hg are lacking.

**Objectives:**

This study aimed to evaluate neurobehavioral changes in mice following combined exposure to Al, Pb, and Hg and to investigate the effects of this exposure on dopaminergic neurotransmission within the striatum.

**Methods:**

In this study, C57BL/6 mice (n = 10 per group) were assigned to control and metal-treated groups. Changes in motor coordination and locomotor activity that occurred when mice were simultaneously exposed to these metals via drinking water for 28 days were measured using the rotarod and open field tests. In addition, dopamine content and key factors involved in dopaminergic neurotransmission in the striatum were evaluated using real-time PCR and Western blot analysis.

**Results:**

The mixed metal exposure decreased motor function and significantly reduced the content of dopamine in the striatum of the experimental mice (P < 0.001). Expression of tyrosine hydroxylase, vesicular monoamine transporter 2, and dopamine receptor D1, which are involved in dopaminergic neurotransmission in the striatum, was significantly decreased (P < 0.01), whereas expression of the dopamine transporter was significantly increased (P < 0.05). Dopamine receptor D2 expression was not significantly changed by the mixed metal exposure.

**Conclusions:**

These results suggest that mixed exposure to Al, Pb, and Hg inhibits normal dopaminergic neurotransmission, resulting in neurobehavioral disorders.

## 1. Background

Industrial developments have increased the use of metals that may be hazardous to human health, resulting in widespread exposure of the general population to toxic metals through polluted drinking water, food, and air (1). Among these toxic metals, aluminum (Al), lead (Pb), and mercury (Hg) are of particular concern due to their high risk of environmental and occupational exposure. They have been identified by the World Health Organization as substances of concern with respect to drinking water and food quality (2-4). These toxic metals enter the human body through various routes and can accumulate in diverse organs, including the brain. The accumulation of toxic metals in the brain can lead to neurodegenerative impairments and neurological disorders (5-7).

Several studies have shown that exposure to Al, Pb, or Hg induces toxicity in the central nervous system and causes neurobehavioral changes in both humans and rodents (8-10). Aluminum exposure occurs through various products, including pharmaceuticals and cooking utensils (11). Studies have shown that such exposure can lead to cognitive dysfunction as well as impairments in learning and memory function (12, 13). Pb is also a significant environmental pollutant. Exposure to high concentrations of Pb is associated with severe damage to the central nervous system, resulting in neurological disorders such as speech impairment and deterioration of motor function (14, 15). Exposure to Hg has been found to interfere with brain development and cause neurobehavioral deficits, including cognitive impairment and limb paresthesia (16).

Al, Pb, and Hg can cross the blood-brain barrier and accumulate in the brain, thereby interfering with the functions of major brain regions and producing adverse neurobehavioral effects (17-20). Exposure to Al, Pb, or Hg commonly induces the generation of reactive oxygen species, leading to the accumulation of oxidative stress and mitochondrial dysfunction (6, 21-24). Furthermore, these metals can cause biochemical dysfunction and loss of dopaminergic neurons in the brain, which is a risk factor for several neurodegenerative diseases (25).

Although numerous studies have investigated the individual neurotoxic effects of these metals, research on mixed exposure to Al, Pb, and Hg remains limited. Exposure to neurotoxic metals can disrupt various neurological processes in the brain, and the dopamine system is one of the primary targets (26). Dopamine, a catecholamine neurotransmitter, plays a crucial role in modulating several central nervous system functions, including motor activity, cognition, reward, attention, and learning (27). Dopamine synthesis is mediated by tyrosine hydroxylase (TH). The synthesized dopamine is then transported and released into the synaptic cleft through vesicular monoamine transporter 2 (VMAT2). The released dopamine binds to postsynaptic dopamine receptors (DRs) to facilitate neurotransmission, while dopamine in the synaptic cleft is reabsorbed into presynaptic neurons via the dopamine transporter (DAT) (26). Dopamine is predominantly distributed in the substantia nigra of the midbrain and is released through the substantia nigra–striatal pathway, playing a crucial role in motor activation within the striatal region (28).

Although numerous studies have investigated the effects of single metal exposure, there is a lack of research on the neurobehavioral effects and dopamine neurotransmission processes associated with mixed metal exposure. 

## 2. Objectives

The present study was performed to assess the neurobehavioral changes in mice following mixed exposure to Al, Pb, and Hg and investigate the impact of this exposure on dopaminergic neurotransmission in the striatum. 

## 3. Methods

### 3.1. Animals

Male C57BL/6 mice (age, 7 weeks; mean weight, 17.73 ± 0.06 g) were obtained from Samtako (Korea) and given unrestricted access to water and food throughout the experimental period. The mice were housed under specific pathogen-free conditions at a temperature of 20°C to 22°C, relative humidity of 55% ± 5%, and a 12-hour light/dark cycle. Aluminum chloride (Alfa Aesar, Ward Hill, MA, USA), lead acetate (Sigma-Aldrich, St. Louis, MO, USA), and methylmercury chloride (Sigma-Aldrich) were procured from commercial suppliers. Body weight, water consumption, feed intake, and clinical symptoms were monitored weekly at 7-day intervals throughout the experimental period, starting from the day of administration.

The metal mixture group received drinking water containing 30 mg/L Al, 25 mg/L Pb, and 10 mg/L Hg. The dosage for each metal was determined based on previous studies (29-31). The control and metal mixture groups comprised 10 animals each, totaling 20 animals used in the study. After 28 days of exposure, the mice were euthanized using carbon dioxide gas, and the striatum was dissected from each animal. The animal experiment was conducted with the approval of the Institutional Animal Care and Use Committee of Keimyung University (KM2022-002).

### 3.2. Neurobehavioral Function

**Figure 1.**
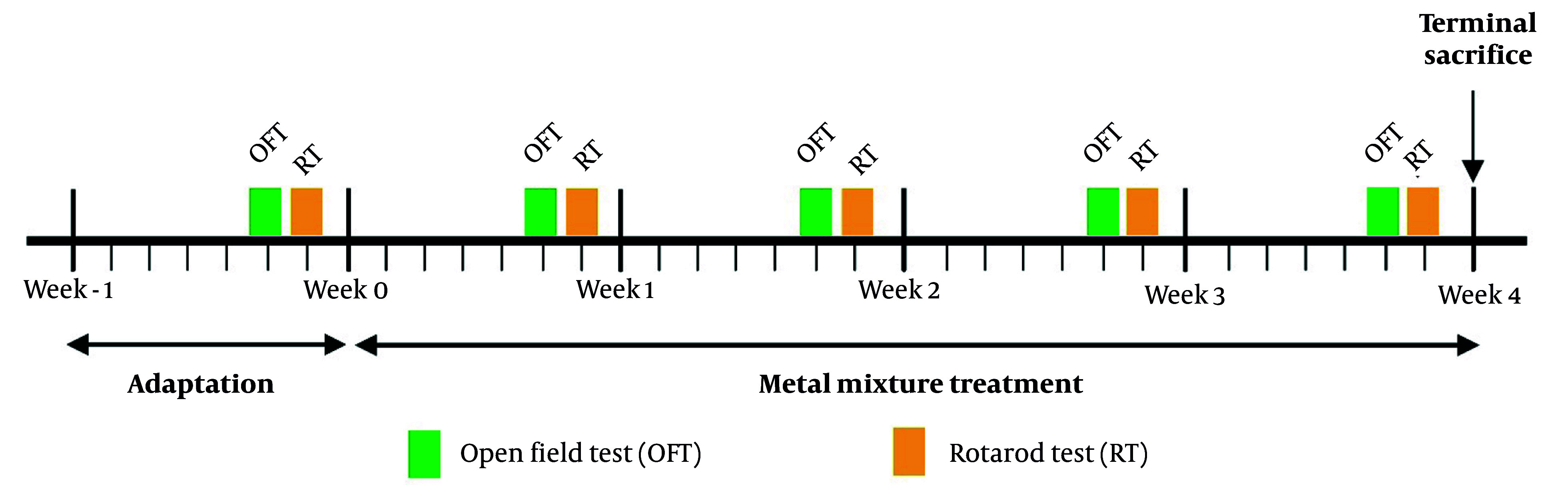
Schematic representation of metal mixture exposure experiment and behavioral test protocol.

Neurobehavioral effects were evaluated by measuring motor coordination and locomotor activity once a week (Figure 1). The rotarod test was employed to assess motor coordination. The test started at a rotation speed of 10 rpm, which was then accelerated over 10 seconds to a maximum speed of 30 rpm. This was repeated three times. The time until falling off (retention time) and the number of slips were recorded during the test, and the average values were used for analysis. The retention time for each experimental group was calculated and expressed as a percentage relative to the retention time before treatment.

In addition, the open field test was used to measure locomotor activity. During the exposure period, each mouse was placed in the center of a square cage (50 × 50 cm) and allowed to freely explore the cage environment for 5 minutes. The SMART video tracking system was used to measure movement and exploration via a camera in the open field test.

### 3.3. Dopamine Measurement

The striatum tissues were collected immediately after euthanasia, briefly rinsed in 1X PBS to remove residual blood, and homogenized in 1 mL of 1X PBS. The homogenized samples were stored overnight at -20°C. To break the cell membranes, two freeze-thaw cycles were performed. The homogenates were then centrifuged at 5000 × g for 5 minutes at 2~8°C. The supernatants were collected and used for analysis.

Dopamine concentrations in the striatum of the mice in the control and metal mixture groups were measured using a dopamine enzyme-linked immunosorbent assay kit (Cusabio, Carlsbad, CA, USA) in accordance with the manufacturer’s instructions. The absorbance of each sample was measured at a wavelength of 450 nm using a microplate reader (Infinite 200 PRO; Tecan, Männedorf, Switzerland). The measurement detection range was set at 5 to 1000 pg/mL with a sensitivity of 2.5 pg/mL.

### 3.4. Real-time Quantitative Polymerase Chain Reaction

For mRNA quantification, total RNA was extracted from the striatum using the NucleoSpin RNA kit (Macherey-Nagel, Düren, Germany) in accordance with the manufacturer’s protocol. RNA samples were quantified using a NanoDrop 2000 spectrophotometer (Thermo Scientific, Waltham, MA, USA), and cDNA was synthesized from the total RNA of each sample using an iScript cDNA Synthesis Kit (Bio-Rad, Hercules, CA, USA).

The quantitative PCR reaction involved the forward and reverse primers shown in Table 1 and was performed using the SsoAdvanced Universal SYBR Green Supermix kit (Bio-Rad) on a CFX96 real-time PCR system (Bio-Rad). The following genes were analyzed, with β-actin used for normalization: Tyrosine hydroxylase (Th, NM_009377.1), dopamine transporter (Dat, NM_010020.3), vesicular monoamine transporter 2 (Vmat2, NM_172523.3), dopamine receptor D1 (Drd1, NM_010076), and dopamine receptor D2 (Drd2, NM_010077), as well as the reference gene β-actin (NM_007393).

**Table 1.** Primer Sequences (5′ to 3′) and NCBI References

Gene	NCBI Ref Seq Number	Sense	Antisense
***Th***	NM_009377.2	GCACATTTGCCCAGTTCTCC	GTACACCGGCTGGTAGGTTT
***Dat***	NM_010020.3	TTGCAGCTGGCACATCTATC	ATGCTGACCACGACCACATA
***Vmat2***	NM_172523.3	ATGTGTTCCCGAAAGTGGCA	AAGTTGGGAGCGATGAGTCC
***Drd1***	NM_010076.3	TAAGCCACCGGAAGTGCTTT	AAGGACCCAAAGGGCCAAAA
***Drd2***	NM_010077.3	AGTGAACAGGCGGAGAATGG	TAGACCGTGGTGGGATGGAT
***β-actin***	NM_007393.5	GCAGGAGTACGATGAGTCCG	ACGCAGCTCAGTAACAGTCC

Data were analyzed using Livak's method (ΔΔCq method), and the expression levels of amplified genes were quantified using CFX Manager Software (Bio-Rad).

### 3.5. Western Blot Analysis

The striatum was homogenized in RIPA homogenizing buffer supplemented with 1% protease inhibitor cocktail (Sigma-Aldrich) to obtain a lysate. The lysate was centrifuged for 20 minutes at 4°C, and the supernatant was collected. Protein concentration was measured using the Bradford method with bovine serum albumin as the standard. A 5-μg protein sample was loaded onto a 10% sodium dodecyl sulfate-polyacrylamide gel electrophoresis (SDS-PAGE) gel and transferred to a nitrocellulose membrane.

The following primary antibodies were used for immunoblotting: Mouse anti-TH monoclonal antibody (diluted 1:150,000; Chemicon, Temecula, CA, USA), rat anti-DAT monoclonal antibody (diluted 1:1000; Santa Cruz Biotechnology, Dallas, TX, USA), mouse anti-VMAT2 monoclonal antibody (diluted 1:1000; Santa Cruz Biotechnology), mouse anti-DRD1 monoclonal antibody (diluted 1:500; Invitrogen, Carlsbad, CA, USA), and mouse anti-DRD2 monoclonal antibody (diluted 1:1000; Santa Cruz Biotechnology).

The immunoreactive signal was detected using an enhanced chemiluminescence reagent and quantified with an LAS 4000 system after incubation with secondary antibodies. The band optical density was analyzed using ImageJ software (National Institutes of Health, Bethesda, MD, USA).

### 3.6. Statistical Analysis

Body weight, neurobehavioral function indices, mRNA expression, and protein expression are presented as mean ± standard deviation (SD). Differences between the control group and the metal mixture group were statistically analyzed using Student’s *t*-test. All statistical analyses were performed using SAS version 9.4 statistical software (SAS Institute Inc., Cary, NC, USA).

## 4. Results

### 4.1. Body Weight

**Figure 2.**
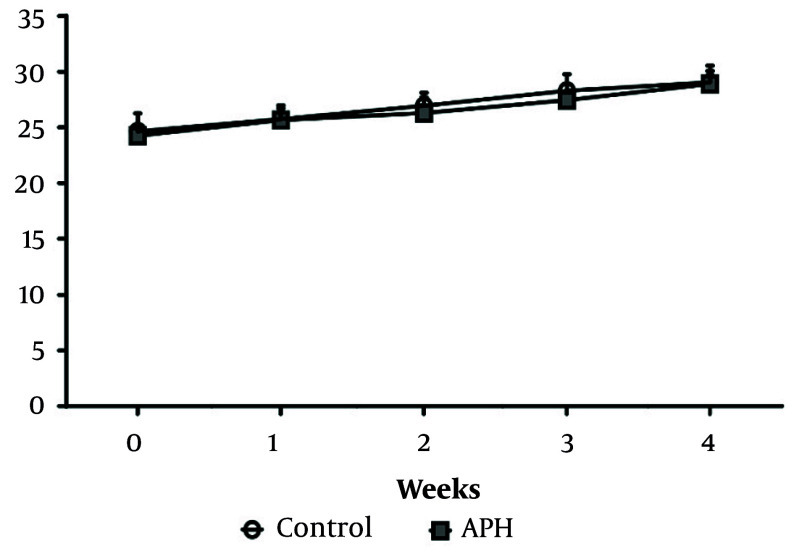
Body weight change during the experimental period. Each value is presented as mean ± SD (n = 10). APH: 30 mg/L Al + 25 mg/L Pb + 10 mg/L Hg.

Throughout the experimental period, no significant differences in body weight were observed between the control group and the metal mixture group (Figure 2). Additionally, there were no significant differences in drinking water or feed intake between the control group and the metal mixture group, and no remarkable clinical signs of toxicity were found in the animals of either group.

### 4.2. Neurobehavioral Function

**Figure 3.**
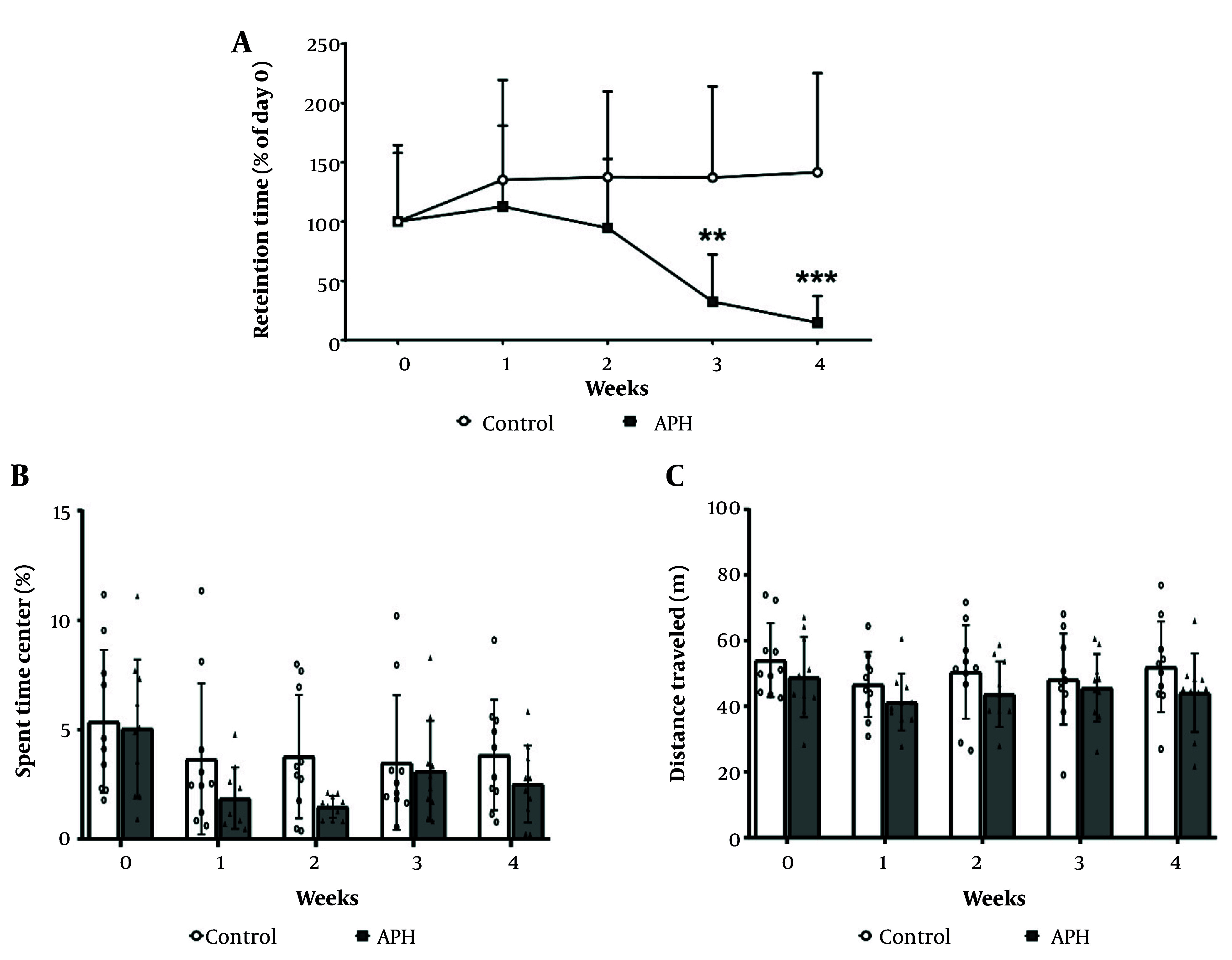
Motor coordination and locomotor activity of mice. A, Motor coordination in C57BL/6 mice during the experimental period. Locomotor activity of the mice during the experimental period was measured and expressed as (B) the time spent in the center; and (C) the total distance traveled. Each value is presented as mean ± SD (n = 10). ** P < 0.01, *** P < 0.001 compared with the control group. APH: 30 mg/L Al + 25 mg/L Pb + 10 mg/L Hg.

The results of the motor coordination assessment using the rotarod test are presented in Figure 3A. Compared with the control group, the metal mixture group exhibited a statistically significant decrease in motor coordination. At 3 weeks of treatment, the motor coordination of the metal mixture group had decreased by 32.4% compared with the control group (P < 0.01), and at 4 weeks of treatment, it had decreased by 14.7% (P < 0.001). However, in the open field test, no significant differences in locomotor activity were observed between the control group and the metal mixture group throughout the experimental period (Figure 3B and C).

### 4.3. Dopamine Concentration in the Striatum

**Figure 4.**
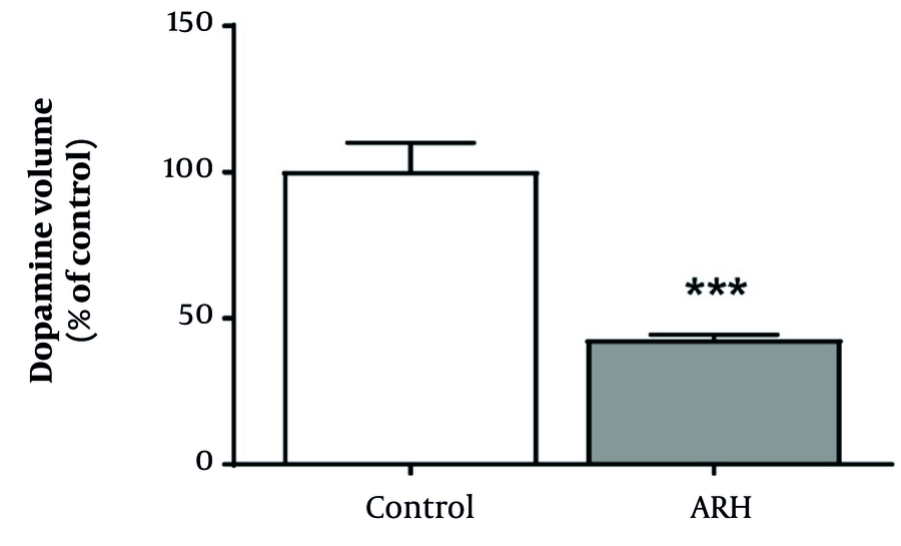
Dopamine volume in the striatum of mice treated with Al, Pb, and Hg. Values are presented as mean ± SD of each group (n = 5). *** P < 0.001 compared with the control group. APH: 30 mg/L Al + 25 mg/L Pb + 10 mg/L Hg.

Figure 4 shows the results of the analysis of the effect of mixed metal exposure on dopamine levels in the striatum. Compared with the control group, the metal mixture group exhibited a statistically significant decrease in dopamine levels (P < 0.001).

### 4.4. Gene Expression in the Striatum

**Figure 5.**
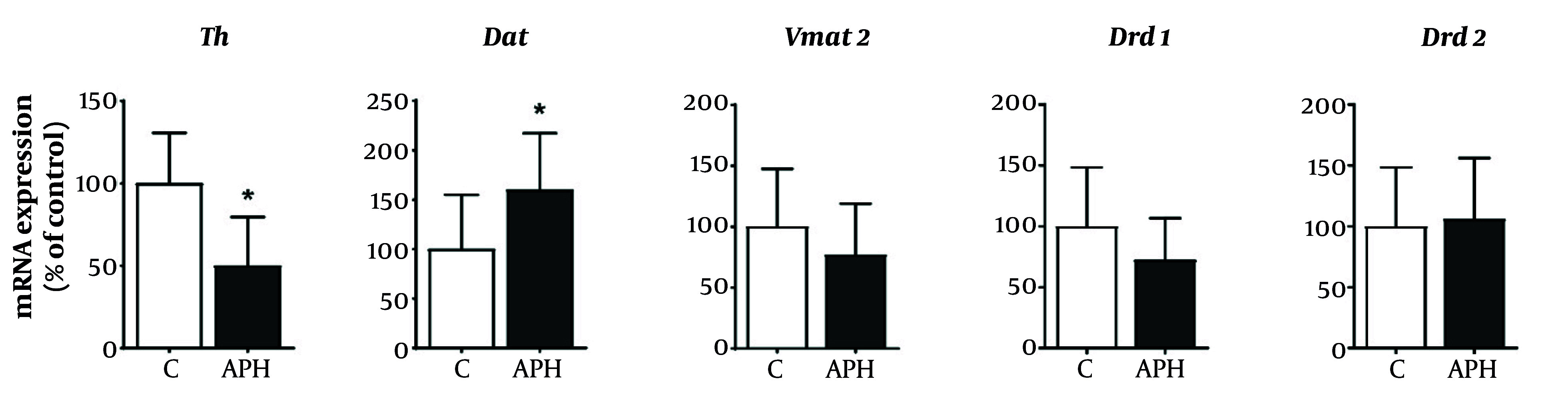
Relative expression of genes involved in dopaminergic neurotransmission in the striatum of mice. Gene expression was normalized against β-actin mRNA expression, and P-values were calculated relative to the control group. Each value is presented as mean ± SD (n = 5). * P < 0.05 compared with the control group. Th: Tyrosine hydroxylase, Dat: dopamine transporter, Vmat2: Vesicular monoamine transporter 2, Drd1: Dopamine receptor D1, Drd2: dopamine receptor D2, C: Control, APH: 30 mg/L Al + 25 mg/L Pb + 10 mg/L Hg.

In the striatal region, the mRNA expression results of genes related to dopaminergic neurotransmission are depicted in Figure 5. Compared to the control group, the metal mixture group exhibited a significant decrease in the gene expression of Th, while the gene expression of Dat in the metal mixture group showed a significant increase (P < 0.05). Furthermore, the metal mixture group exhibited a decreasing trend in the gene expression of Vmat2 and Drd1 compared to the control group. However, no significant difference was observed in the expression of Drd2 between the metal mixture group and the control group.

### 4.5. Protein Expression in the Striatum

**Figure 6.**
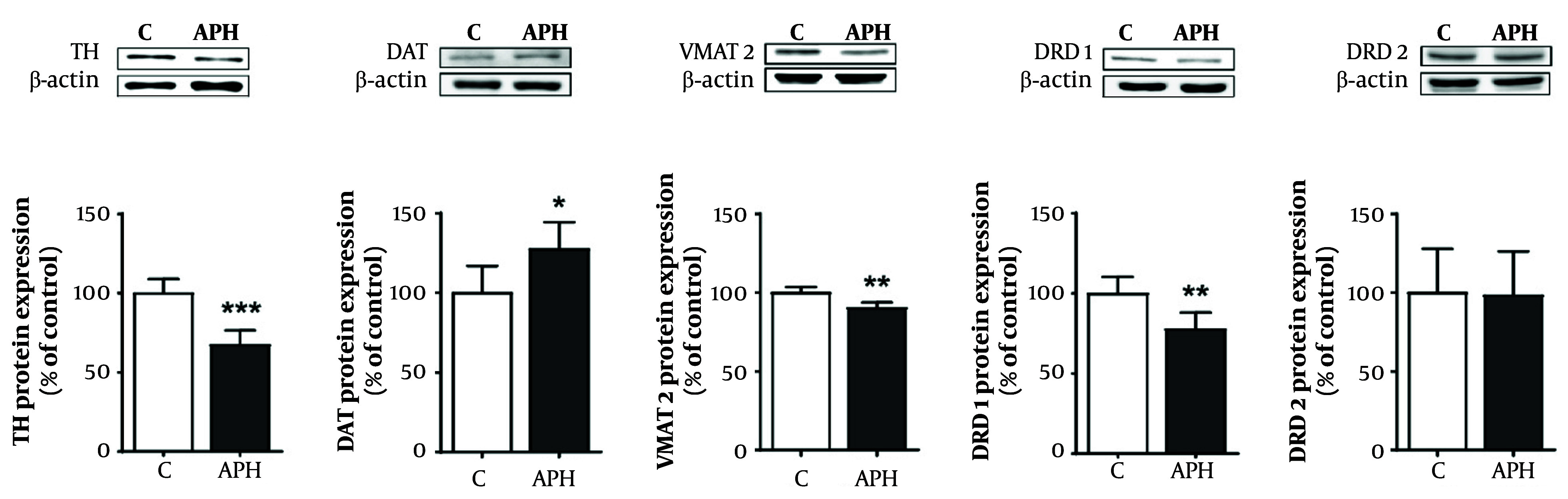
Relative protein expression related to dopaminergic neurotransmission in the striatum of mice. Each value is presented as mean ± SD (n = 5). * P < 0.05, ** P < 0.01, and *** P < 0.001 compared with the control group. TH: Tyrosine hydroxylase, DAT: Dopamine transporter, VMAT2: vesicular monoamine transporter 2, DRD1: Dopamine receptor D1, DRD2: Dopamine receptor D2, C: Control, APH: 30 mg/L Al + 25 mg/L Pb + 10 mg/L Hg.

Similar to the gene expression results, the protein expression of TH, VMAT2, and dopamine receptor D1 (DRD1) significantly decreased in the striatum of the metal mixture group compared with the control group (P < 0.01 or P < 0.001). However, the protein expression of DAT significantly increased in the metal mixture group compared with the control group (P < 0.05). Moreover, consistent with the gene expression results, there was no significant difference in dopamine receptor D2 (DRD2) protein expression between the metal mixture group and the control group (Figure 6). 

## 5. Discussion

The present study was conducted to investigate the neurotoxic effects of mixed exposure to the toxic metals Al, Pb, and Hg. Metal pollution caused by population growth and industrial and agricultural development is a major cause of water quality problems, and contaminated water can be absorbed into the human body, leading to various health issues (32, 33). Most studies on the effects of metal exposure in the human body have been conducted under single-metal exposure conditions; there is a lack of information on exposure to metal mixtures. Therefore, this study was performed to evaluate the neurobehavioral effects that can occur due to exposure to a metal mixture, and the results confirmed the relationship between these neurobehavioral effects and dopaminergic neurotransmission.

To assess the motor function impairments that may occur due to mixed metal exposure, we measured motor coordination and locomotor activity of mice using the rotarod test and open field test, respectively. The rotarod test showed a significant decrease in motor coordination in the metal mixture group compared with the control group after 3 weeks of metal administration. At the fourth week of administration, the motor coordination of the metal mixture group had decreased to 14.7% of its baseline measurement at week 0. Comparison of our results with previous studies that investigated the effects of single exposure to Al, Pb, or Hg (34-36) indicates that exposure to the metal mixture in our study led to a more pronounced reduction in motor coordination.

Previous studies have also shown that single-metal exposure to Al, Pb, or Hg can decrease locomotor activity (37-39). In our study, however, although the locomotor activity in the metal mixture group exhibited a decreasing trend, it did not reach statistical significance when compared with the control group. This suggests that the effects of exposure to a metal mixture on locomotor activity may vary depending on the exposure duration, dose level, and exposure route. Taken together, the results of our study suggest that exposure to a metal mixture may lead to motor function impairment in mice.

Dopaminergic neurons in the midbrain provide a dense neural projection to the striatum and are involved in motor and learning functions (28, 40). Previous studies have shown that motor activity is partially determined by the activity of the striatum (41). Therefore, the changes in motor function caused by exposure to a metal mixture are closely related to the abnormalities in the dopaminergic neurotransmission process in the striatum. Our study also revealed a significant reduction in dopamine levels within the striatum of mice exposed to the metal mixture when compared with the control group. This result indicates that exposure to a metal mixture can lead to depletion of dopamine, thereby causing a disturbance in dopaminergic neurotransmission.

TH is a rate-limiting enzyme in dopamine synthesis and a key enzyme that determines dopamine levels (42). TH gene and protein expression in the striatum was significantly decreased in mice exposed to the metal mixture. This result is consistent with the results of previous studies showing that Al, Pb, or Hg can affect TH expression (37, 43, 44). In addition, we investigated gene and protein expression of VMAT2 and DAT, which play roles in dopamine transport and reuptake, respectively, in the dopaminergic neurotransmission process. VMAT2 gene and protein levels were downregulated in the striatum of mice exposed to the metal mixture. This result is consistent with the results of previous studies showing that Pb exposure decreased VMAT2 expression (45). Decreased VMAT2 expression has also been reported in mice exposed to metal mixtures, including arsenic and Pb (46, 47). The present study indicates that mixed exposure to Al, Pb, and Hg may impact the expression of VMAT2, potentially leading to inhibition of dopamine transport.

By contrast, DAT gene and protein levels were upregulated in the striatum of mice exposed to the metal mixture. This result is consistent with the results of previous studies showing that DAT expression can be increased in mice exposed to Al, Pb, or Hg (48, 49). Increased DAT expression may occur to compensate for the decrease in TH and VMAT2 expression caused by metal exposure (50).

Dopamine receptors (DRs) are classified into two subfamilies based on their biochemical and pharmacological characteristics: D1-like and D2-like receptors. Dopamine D1 and D2 receptors are primarily expressed in the striatum and reflect dopamine levels (51). Therefore, we investigated the expression of DRD1 and DRD2 in the striatum of mice exposed to the metal mixture. Our results showed that DRD1 gene expression tended to decrease, whereas protein expression significantly decreased. Previous studies have shown that single-metal exposure to Al, Pb, or Hg decreases DRD1 expression in mice (52-54). However, we found no significant difference in the expression of the DRD2 gene or protein in the striatum of mice exposed to the metal mixture compared with the control group.

DRD2 is linked to inhibitory G proteins (Gi), while DRD1 is associated with stimulatory G proteins (Gs). Therefore, Al, Pb, and Hg (APH) are more likely to influence DRD1-associated pathways (via Gs proteins), leading to an increase in cAMP levels, rather than affecting DRD2-linked Gi pathways that typically inhibit cAMP production. Dopamine plays a critical role in regulating motor coordination in the striatum, and DRD1 is closely related to the regulation of motor coordination and balance (55). Therefore, the previously reported decrease in DRD1 expression caused by mixed metal exposure is consistent with the results of motor function in the present study, suggesting that DRD1 plays an important role in the neurobehavioral abnormalities caused by mixed metal exposure.

This study has several limitations. The 28-day oral exposure period set in this research may not fully reflect the long-term effects of chronic mixed metal exposure on dopaminergic neurotransmission and neurobehavioral outcomes. Al, Pb, and Hg exhibit high bioaccumulation, with half-lives in the brain ranging from several months to years, suggesting their effects on striatal targets could persist over an extended period. To evaluate cumulative effects more comprehensively, further studies incorporating various exposure routes and extended durations are necessary. Additionally, the combination of metals used in the experiment may not fully represent the levels and compositions of actual environmental exposure. Exploring a broader range of concentrations and combinations would help enhance the generalizability of the results.

In conclusion, this study demonstrated that mixed exposure to Al, Pb, and Hg caused disturbances in motor coordination and decreased dopamine levels in the striatum. Additionally, the abnormalities in dopaminergic neurotransmission, which are caused by alterations in the expression of TH, VMAT2, DAT, and DRD1 in the striatum, were found to be closely associated with the neurobehavioral effects caused by exposure to the metal mixture. Further research is warranted to investigate the molecular mechanisms of neurotransmission changes induced by mixed metal exposure, such as oxidative stress, mitochondrial dysfunction, and epigenetic modifications.

## Data Availability

The data presented in this study are available on request from the corresponding author.
